# A new biosensor for noninvasive determination of fetal RHD status in maternal blood of RhD negative pregnant women

**DOI:** 10.1371/journal.pone.0197855

**Published:** 2018-06-06

**Authors:** Ebru Dündar Yenilmez, Umut Kökbaş, Kezban Kartlaşmış, Levent Kayrın, Abdullah Tuli

**Affiliations:** Department of Medical Biochemistry, Faculty of Medicine, University of Cukurova, Adana, Turkey; Xavier Bichat Medical School, INSERM-CNRS - Université Paris Diderot, FRANCE

## Abstract

Prenatal detection of the fetal *RHD* status can be useful in the management of RhD incompatibility to identify fetuses at risk of hemolytic disease. Hemolytic disease causes morbidity and mortality of the fetus in the neonatal period. The routine use of antenatal and postnatal anti-D prophylaxis has reduced the incidence of hemolytic disease of the fetus and newborn. This study describe the detection of fetal RhD antigens in blood of RhD negative pregnant women using a nanopolymer coated electrochemical biosensor for medical diagnosis. Cell free fetal DNA in maternal plasma was also used to genotyping fetal *RHD* status using multiplex real-time PCR. Twenty-six RhD negative pregnant women in different gestational ages were included in the study. RhD positive fetal antibodies detected with a developed biosensor in maternal blood of RhD negative mothers. The electrochemical measurements were performed on a PalmSens potentiostat, and corundum ceramic based screen printed gold electrode combined with the reference Ag/AgCl electrode, and the auxiliary Au/Pd (98/2%) electrode. Fetal *RHD* genotyping performed using fluorescence-based multiplex real-time PCR exons 5 and 7 of the *RHD* gene. The fetal *RHD* status of 26 RhD negative cases were detected 21 as RhD positive and 5 as RhD negative with electrochemical biosensor. Fetal *RHD* status confirmed with extracted fetal DNA in maternal plasma using multiplex real-time PCR *RHD* genotyping and by serological test after delivery. The new method for fetal RhD detection in early pregnancy is useful and can be carry out rapidly in clinical diagnosis. Using automated biosensors are reproducible, quick and results can be generated within a few minutes compared to noninvasive fetal *RHD* genotyping from maternal plasma with real-time PCR-based techniques. We suggest the biosensor techniques could become an alternative part of fetal *RHD* genotyping from maternal plasma as a prenatal screening in the management of RhD incompatibility.

## Introduction

The cause of Rhesus hemolytic disease (RhD) of the RhD positive fetus of RhD negative pregnant women is the maternal IgG antibodies produced against the RhD antigen of the fetal erythrocytes. This situation has the significant cause of morbidity and mortality for the fetus. In order to prevent fetal hemolysis, the routine use of antenatal and postnatal prophylaxis with anti-RhD immunoglobulin, substantially reduces the alloimmunization of RhD negative women. [1–5].

Prenatal determination of the fetal *RHD* genotyping can be useful in the management of RhD incompatibility [6]. Significant progress in prenatal care strategies for the fetus with RhD has occurred during the last few decades. Cell-free fetal DNA (cffDNA) discovered from plasma of pregnant women by Lo et al in 1997 has been used for the noninvasive detection of fetal RhD status [7–9], which has the potential to avoid antenatal anti-RhD prophylaxis in RhD negative women [5, 10–12]. Many obstetricians accept fetal RhD status detection using circulating cffDNA from maternal plasma or serum. [13–18].

Nowadays, biosensors are widely used in different areas of healthcare [19]. Pregnancy test and glucometer are two main examples of very successful biosensor devices. Different transducing mechanisms are employed in immunological biosensors, based on signal generation (such as an electrochemical or optical signal) following the formation of antigen-antibody complexes [20]. High-affinity reagents such as antibodies, enzymes and synthetic biomolecules can be coupled to the transducer in order to provide specificity of the biosensors [21].

In the study, we aimed to design a new nanopolymer coated electrochemical biosensor specific for detection of fetal RhD antigens in blood of pregnant women and the results compared with cffDNA *RHD* genotyping with real-time PCR.

## Materials and methods

The electrochemical measurements were performed on a PalmSens potentiostat (Holland), and corundum ceramic based screen printed gold electrode (tickness 1.0 mm, BVT Technologies, CZ) combined with the reference Ag/AgCl electrode, and the auxiliary Au/Pd (98/2%) electrode. Automatic pipets (Gilson, France), a yellow line magnetic stirrer (Germany), and a thermostat (Nuve, Turkey), were used in the experiments. Ultra-pure water in the preparation of solutions was obtained from water purification system (Mili-Q and Milipore RIOS-DI 3 UV, USA).

### Preparation procedure of the Au electrode surface

#### Cleaning electrode

Prior to coating with nanopolymer, the surface of Au ceramic electrode was polished with alumina slurries on microfiber cloth to obtain a mirror surface. The polished electrode rinsed with double distilled water. In order to remove undesired absorbable particles, the electrode sonicated first in pure ethanol and later in double distilled water for 10 minutes. In the next step, the electrochemical cleaning of electrode was accomplished by five successive cyclic voltammogram sweeps between −1.0 and +1.0 V in 0.1 M HNO_3_ solution.

#### Immobilization of RhD antibody onto Au electrode surface

Nanopolymer (Poly Hema-Mac) was applied on the surface of the clean electrode at room temperature until to the formation of Au-Poly HemaMac electrode. At the end of these periods, immobilization of the RhD antibody was performed on the modified electrode surface with anilin (20μL RhD antibody and 20μL anilin). Finally, for trapping immobilized antibody, the electrode was immersed in solution of crosslinking agent (2.5% glutaraldehyde) for 1 h at UV light for polymerization. The electrode was rinsed with double distilled water and could be stored at 4°C for future use.

#### Electrochemical measurement principle

**Fig 1:**
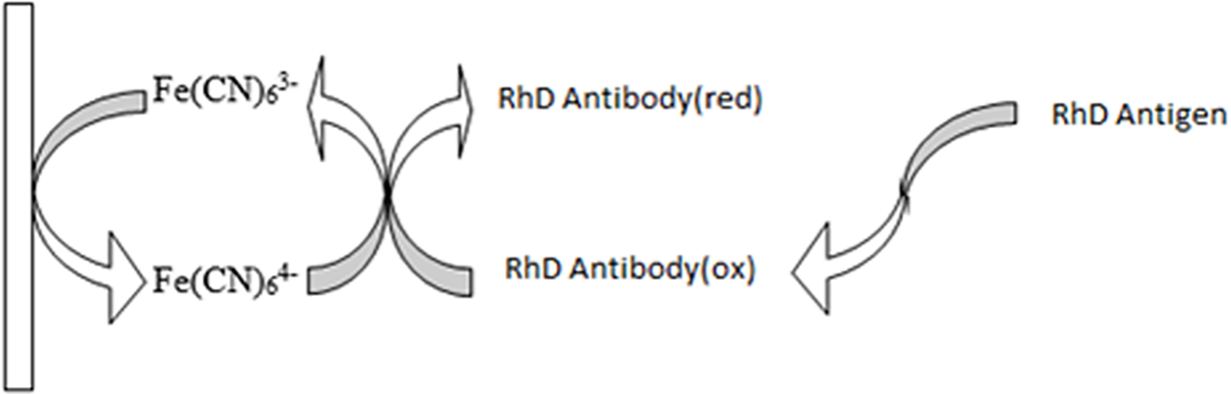
The principle of the biosensor.

The measurement of the biosensor based on the oxidation-reduction reactions put into the thermostatic reaction cell included phosphate buffer (50 mM, pH 7.0) and potassium ferrocyanide [K_4_Fe(CN)_6_] as mediator complex, at 35°C. The difference in charge transfer capacitance (electrochemical potential difference) of antigen-antibody interaction was measured by biosensor (Fig 1).

#### Sample preparation

The study population comprised of 26 RhD negative primigravidas who admitted to the Department of Gynecology and Obstetrics and to the Department of Medical Biochemistry for prenatal diagnosis of hemoglobinopathies in different gestational ages (8^th^-36^th^ weeks) (Table 1). Written informed consent that was approved by the Ethics Committee of the Faculty of Medicine of Cukurova University was obtained from each subject. Blood samples obtained from 26 samples in ethylenediamine-tetraacetic acid (EDTA) tube (Becton Dickinson, Bangkok, Thailand) Blood group test was identified by the Blood Bank Centre using slide/tube agglutination test, which includes antibodies against red blood cell antigens.

**Table 1:** Clinical features of the study population.

Fetal RhD Status	Age (years)		Gestational Age		Immunization
	X ± SD (min-max)	Range	X ± SD (min-max)	Range	
**RhD positive (n = 21)**	29.5 ± 5.9 (19–37)	18	15.1 ± 6.7 (8–36)	28	No
**RhD negative (n = 5)**	25.6 ± 3.2 (21–30)	9	11 ± 2.0 (8–13)	5	No

### Fetal *RHD* genotyping from maternal blood samples

#### Fetal DNA extraction

A 10 mL sample of maternal blood was collected from each pregnant woman and was taken into the tube with EDTA. Within one hour, the maternal plasma was separated by centrifugation at 1600 *g* for 10 minutes. Then, to obtain purified supernatant, the collected plasma in polypropylene tubes was again centrifuged at 16 000 *g* for 10 minutes. The supernatants were stored at –20°C until further processing such as real-time PCR for *RHD* genotyping. The supernatants were thawed and the DNA was automatically extracted from 1 mL of plasma as reported in Yenilmez et al. [22].

#### Genotyping with real-time quantitative PCR of *RHD* gene exon 5 and 7

The isolated 26 cffDNA samples were tested for the presence of the *RHD* gene (exons 5 and 7). Real-time quantitative PCR was performed in Light Cycler 480 (Roche Applied Science, Basel, Switzerland). The PCR reactions were performed in a total volume of 50 μL reaction mixture which was containing 300 nM of each primer, 2×TaqMan Universal PCR master mix (Roche Diagnostics, Basel, Switzerland), 50 nM probe, and 15 μL of template DNA from the samples. The cycling conditions were performed as mentioned by Yenilmez et. al. [22].

## Results and discussion

### RhD antibody immobilization

**Fig 2:**
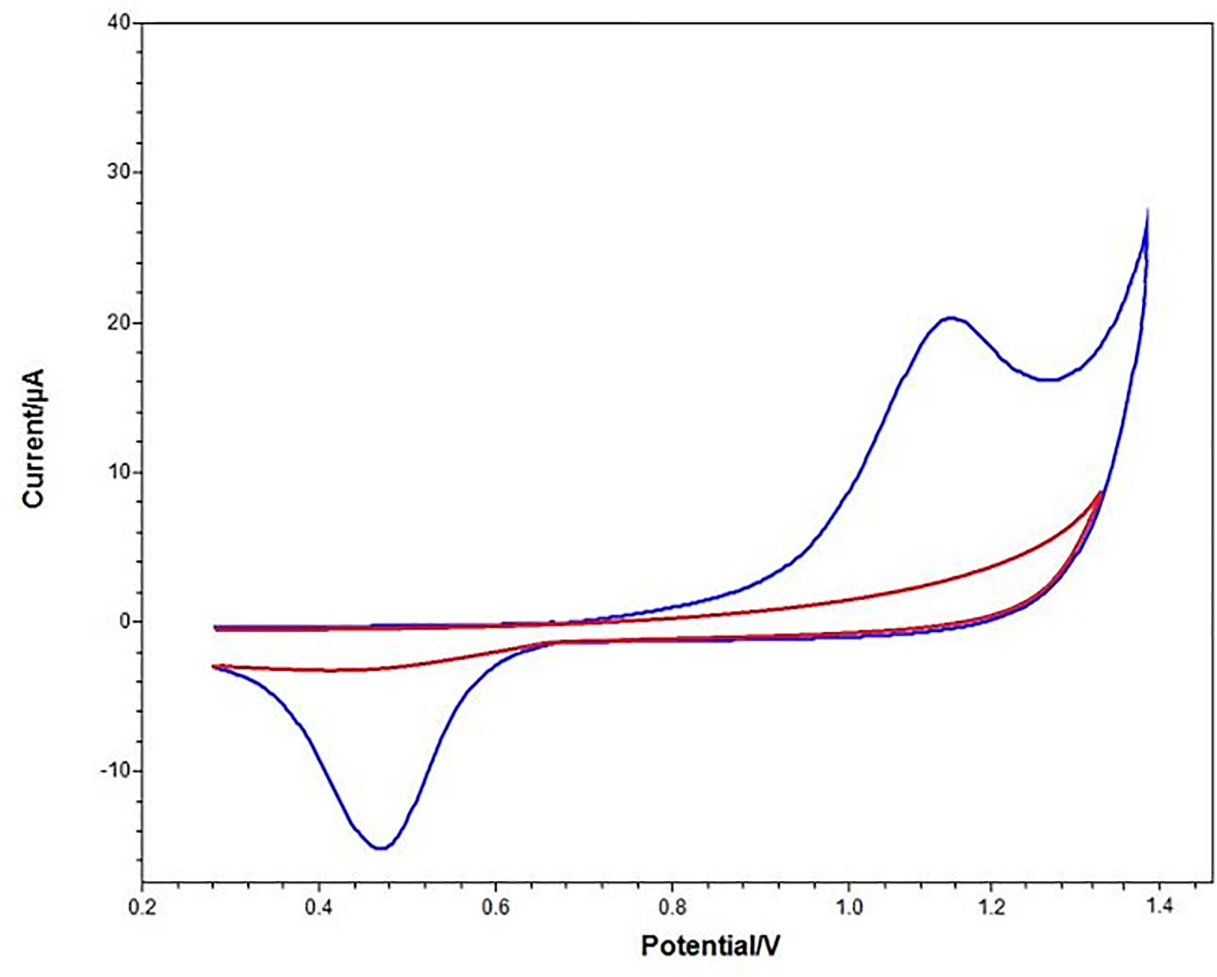
Cyclic voltammogram for the immobilization steps of the RhD biosensor. Red line: bare gold electrode; blue line: after UV polymerization. (Working conditions: Incubation time for RhD antibody: 1 h., electrochemical redox prob solution: 50 mM, pH 7.0 potassium ferrocyanide [K_4_Fe(CN)_6_] as mediator complex). The optimal curve of the biosensor over a potential range from 0.2 to 1.4 V in the detection of RhD antigen in maternal sample.

UV light method were used for immobilization of RhD antibody. The UV light used to reduce the anilin’s reduction potential. The polymerization process perform quickly via UV light. As shown in Fig 2 there is a cyclic voltammogram of the redox probe, Fe (CN)_6_^4-/3-^ showed a reversible manner on the uncovered working electrode. The bioactive layer performed on the surface of electrode inhibited the charge transfer among redox probe in solution and the Au electrode surface. The cyclic voltammogram with reversible behavior turned into a capacitive shape (Fig 2).

### Optimization trials for RhD antibody biosensor

Optimization studies of working situations determine the most suitable working conditions for using the biosensor. For this aim, the RhD antibody and the cross-linker, mediator concentration, the pH and temperature effect and the repeatability were investigated.

#### RhD antibody concentration

To determine the effect of the antibody concentration on the biosensor response, different RhD antibody concentrations (0.05, 0.10, 0.15,0.20 ng/mL) was applied on the surface of biosensor. The optimum concentration of antibody of the bioactive layer on biosensor was determined at 0.10 ng/mL.

#### Cross-linker and mediator concentration

To determine the effect of cross-linker concentration on the biosensor, the concentrations of glutaraldehyde of 12.5% and 2.5% were used. The optimum value obtained at 2.5%. In order to investigate the effect of the mediator concentration on the biosensor response, potassium ferrociyanide of 1.25 mg/dL and 2.5 mg/dL were used in the preparation of the biosensor. According to the results obtained from the experiments, the mediator complex of 1.25 mg/dL was assigned the most effective results for the biosensor.

#### pH effect

Biosensors based on an antibody depends on a suitable buffer system and pH medium for obtaining the best responses. To detect the effect of the pH value on the biosensor response, different buffer systems were investigated. For this aim, acetate (50 mM, pH 5.0–5.5), phosphate (50 mM, pH 6.0–6.5–7.0–7.5), and Tris-HCl (50 mM, 8.0–8.5) buffers were used in the experiments. The optimum pH value was 7.0 due to 100% activity rate. Below and above pH 7.0 causes a decrease in the biosensor response.

#### Temperature effect

**Fig 3:**
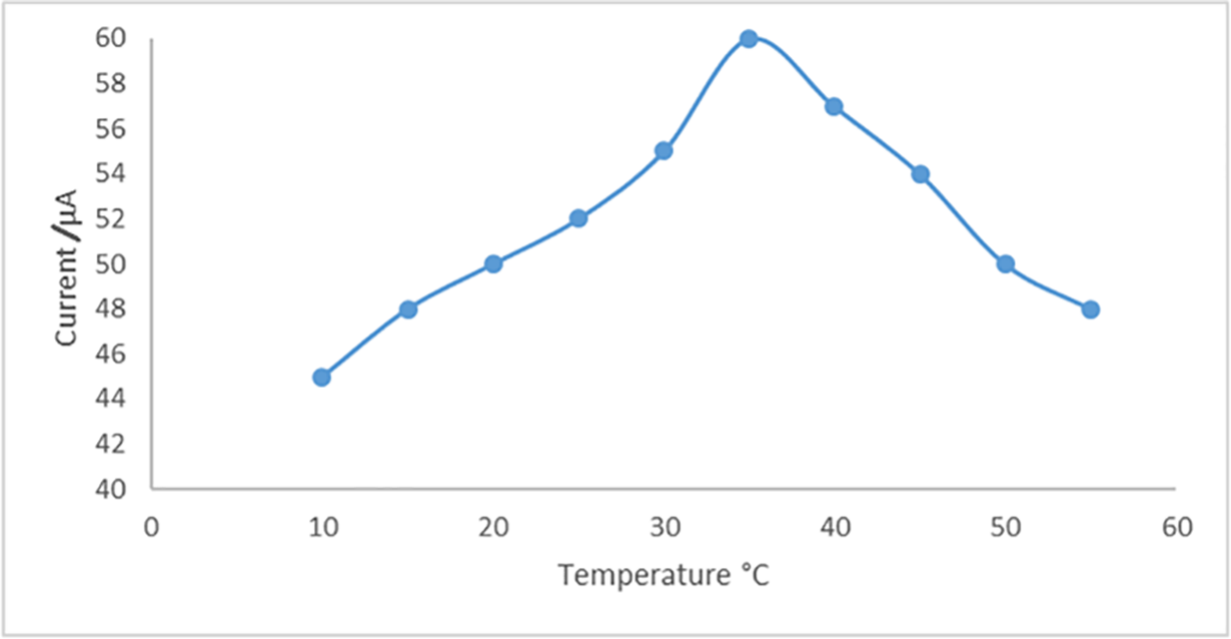
The optimum working temperature of the biosensor. Optimum working temperature of the biosensor was detected as 35°C (Working conditions: Incubation time for RhD antibody: 1 h., electrochemical redox prob solution: 50 mM, pH 7.0 potassium ferrocyanide [K_4_Fe(CN)_6_] as mediator complex).

For the determination of temperature effect on the biosensor response, the assay was performed by different temperatures (10–55°C). Optimum working temperature of the biosensor was detected as 35°C. The biosensor response directly increased with temperature until 35°C (Fig 3), but further increase in temperature caused a decrease on the biosensor response.

#### Repeatibility

Determination of the repeatability of the biosensor experiments were also studied for 1 μM RhD concentration (n = 10). From the assays the mean value ($\bar{X}$), standard deviation (SD) and coefficient of variation (CV %) were found to be 2.68±0.06 μM, and 2.23%, respectively. From results, the repeatability of the biosensor response can be accepted as well within given concentration of RhD according to the 95% confidence interval.

### Characterization of RhD antibody biosensor

**Fig 4:**
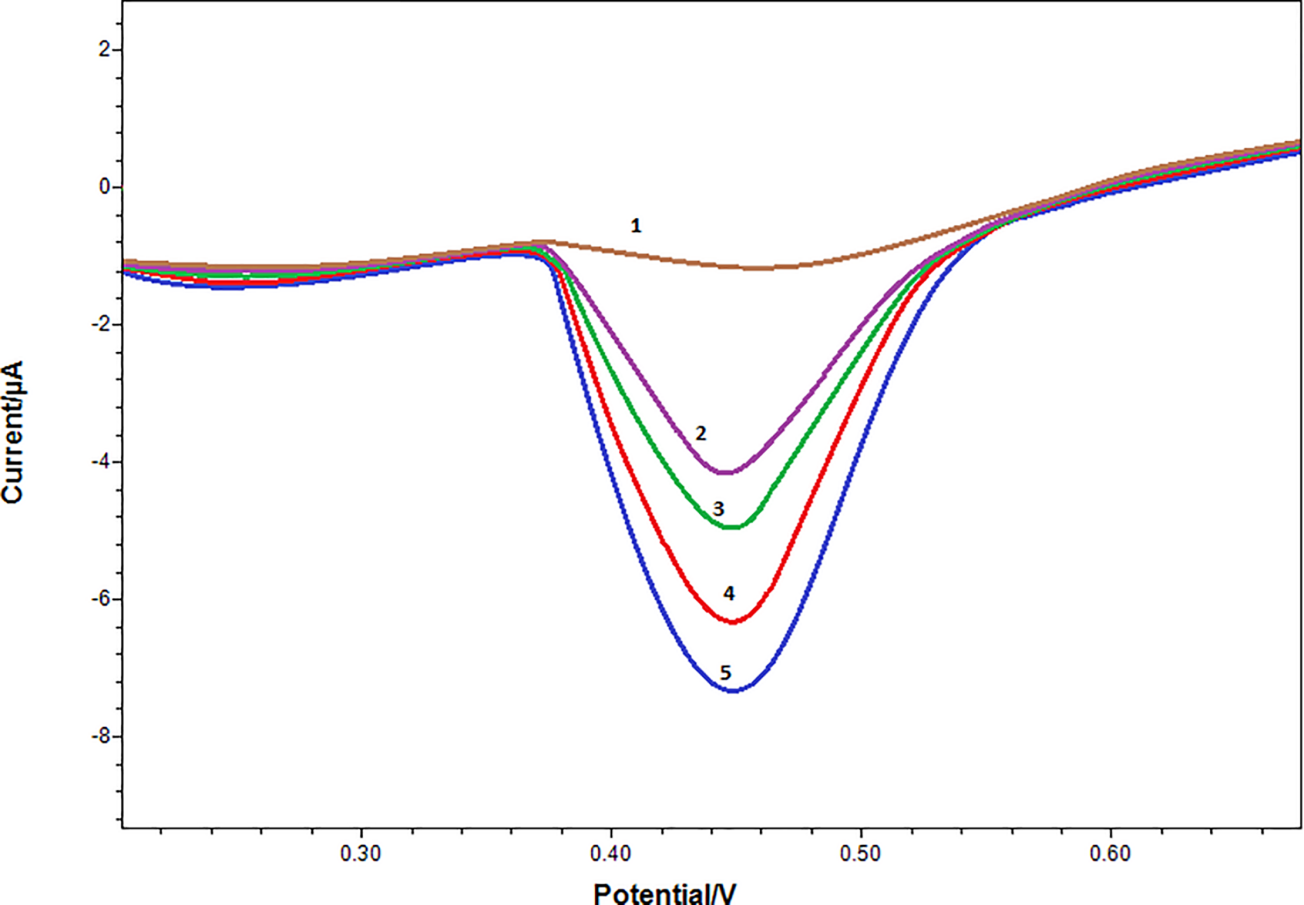
Detection of increasing fetal RhD antigen with biosensor in different gestational age mother’s blood. Curve 1(brown): RhD negative sample; Curve 2(purple): sample 8^th^ week of gestation; Curve 3(green): sample 13^th^ week of gestation; Curve 4(red): sample 21^th^ week of gestation; Curve 5(blue): sample 36^th^ week of gestation. (Working conditions: Incubation time for RhD antibody: 1 h., electrochemical redox prob solution: 50 mM, pH 7.0 potassium ferrocyanide [K_4_Fe(CN)_6_] as mediator complex).

Fig 4 shows the graphic for RhD concentrations in different gestational ages of pregnant women samples. The curves increased with the increasing fetal RhD antigen concentration depend on gestational ages of the samples (Fig 4).

**Fig 5:**
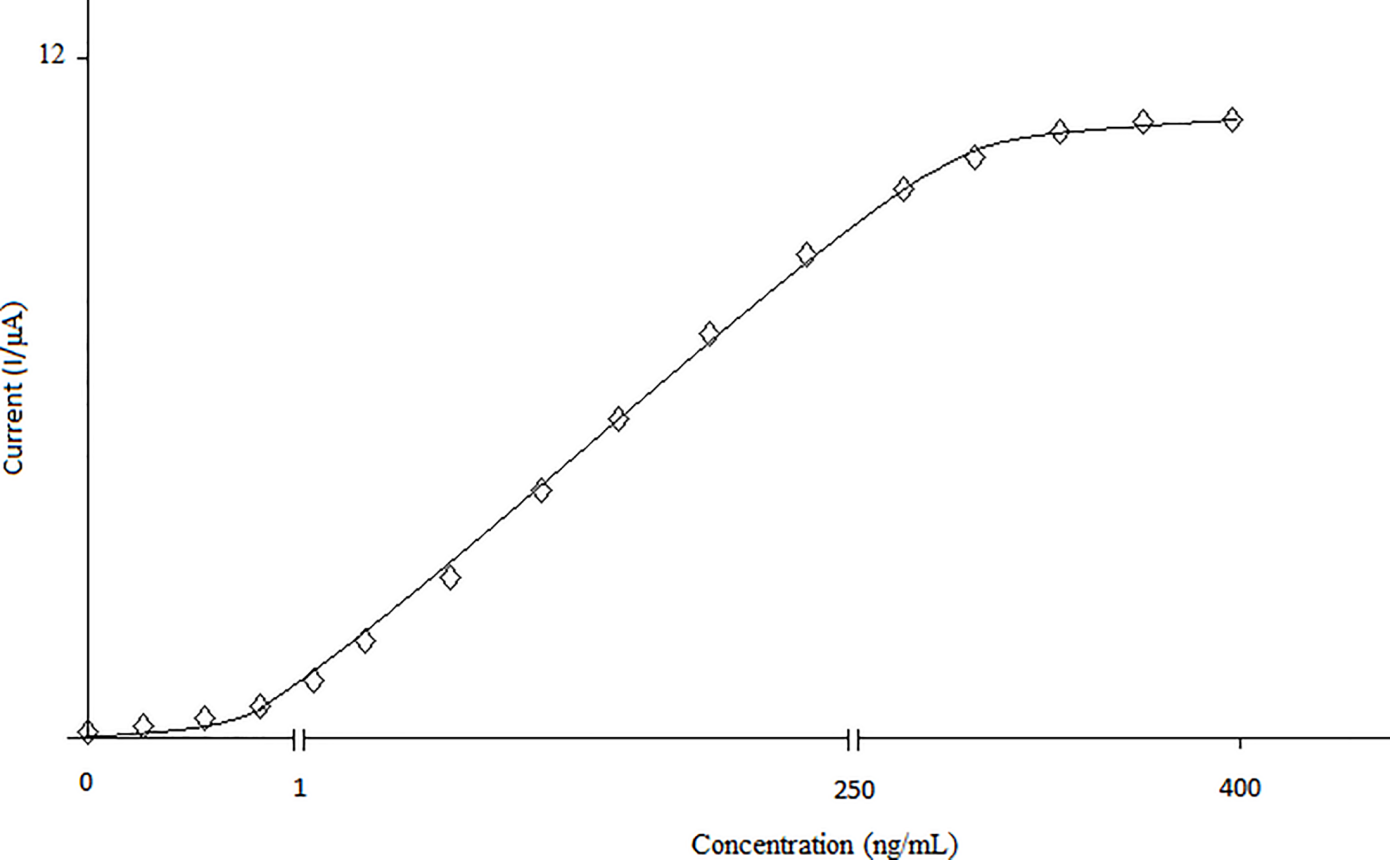
The linearity of biosensor antibody. (Working conditions: Incubation time for RhD antibody: 1 h., electrochemical redox prob solution: 50 mM, pH 7.0 potassium ferrocyanide [K_4_Fe(CN)_6_] as mediator complex).

The linearity study for the RhD biosensor was obtained in concentration range between 1 to 250 ng/mL (Fig 5). At higher concentrations, standard curve showed a deviation from linearity (Fig 5).

### Fetal RHD genotyping

The fetal RhD status of the fetus from fetal DNA verified in 26 pregnancies with multiplex real-time PCR for *RHD* gene exon 5 and 7. Twenty-one cffDNA results were detected RhD positive and 5 were RhD negative (The same results as detection with RhD biosensor). The results of the fetuses confirmed also by serological and molecular tests with fetal DNA genotyping after delivery.

## Conclusion

This study showed a biosensor design, which detects RhD status of the fetus in the early stage of pregnancy in RhD negative pregnant women blood. RhD antibody immobilized using UV polymerization of anilin. In the literature, not this kind of biosensor system have been reported before.

Impedence measurements were applied to characterize the electrochemical properties of the biosensor surface. And also we showed the stable bioactive layer was formed for binding of RhD antigen of fetus. The antigen-antibody binding resulted in significant impedance response that was detected even at concentration of 1 ng/mL RhD. This results were confirmed with real-time PCR fetal RHD genotyping of the fetus.

The biosensor system based on antigen-antibody detection has more advantage to detect the RhD status of the fetus fast at least as early as the noninvasive fetal *RHD* genotyping using fetal DNA. Up to now, most common methods for the determination of fetal RhD status were based on serological techniques on delivery. Clinical and molecular diagnostic studies require fast, sensitive and low cost techniques. The discovery of fetal DNA in maternal plasma has opened up new and exciting opportunities for detection of the fetal blood group status using NIPD [23]. The noninvasive technique based on the analysis of fetal RhD status of cffDNA with qPCR have been recently introduced and now is a strong alternative in early pregnancy. The advantage of NIPD is the early detection of RhD status and avoid the mother from unnecessary anti-RhD prophylaxis [24, 25]. Extraction fetal DNA in maternal plasma is a better way to determination fetal *RHD*. The last decade there were important developments in the correct management of pregnancies in not immunized and/or alloimmunized RhD negative pregnant women by noninvasive fetal *RHD* genotyping [26, 27]. The fetal nucleated red blood cells (RBCs) are present in maternal blood is well known [28]. Bianchi et al. revealed that nucleated RBCs are abundant in first-trimester fetal blood, during the yolk sac and liver phases of haemopoiesis and the erythrocyte line develops earlier in gestation than the white cell line [29]. The RhD antigen is expressed on the RBC membrane, and alloimmunization can be caused when fetal RhD-positive RBCs enter maternal circulation, and the RhD-negative mother develops anti-D antibodies.

RhD antigen belongs to fetus can be detected about 30-40^th^ day of pregnancy. The 21 RhD positive fetuses created signals (the signals increased in proportion to the gestational week) which meant that the fetal RhD antigens on fetal RBCs bind on the surface of the biosensor that coated with RhD antibodies. The formed complex (antigen-antibody) generates a chemical signal (expressed in the graphic), which are converted into an electrical signal by means of a transducer in RhD positive fetuses. In five of our samples the fetus was RhD negative and there was no change of the signal of biosensor.

This study exposes a new, quick, reliable and easy detection of fetal RhD positive antigens from RhD negative pregnant women blood using a biosensor. This immunospecific biosensor offers an alternative noninvasive prenatal detection method for fetal RhD status to management of RhD incompatibility. The developed biosensor assay for RhD antigen, is able to capture fetal RhD antigens in maternal blood of RhD in early stages of pregnancy (8^th^ week of pregnancy).

The fetal RhD status detecting with biosensor takes several minutes using a gold electrod covered by RhD antibody. The novel biosensor is more suitable especially for routine fetal RhD determination in early pregnancy because it is simple to construct and sensitive, specific and does not require any expensive apparatus. The biosensor devices exhibits low cost with regard to real-time PCR instruments. The biosensors can be used again for several times (up to 400 fold), and so the cost decreases.

Currently, the most frequently used technique for NIPD is the qRT-PCR. There are biosensor studies reported that NIPD application with cffDNA for monogenic diseases [30]. Brevegileri et al. published Y-chromosome detection in cffDNA with Surface plasmon resonance (SPR) based biosensors [31]. Some studies described PCR-free applications using SPR-imaging [32]. We prepared a study for detecting fetal RHD genotyping from cffDNA using SPR based biosensor. The proposed biosensor was specific for RhD antigen, and could be design to use in the detection of other antigen-antibody studies. In addition, the ability of less sample use, lower cost, and the ability to determine fetal RHD status in a very short time makes the biosensor more advantages than NIPD of RhD using real-time quantitative PCR.
